# MiR-195 suppresses the metastasis and epithelial–mesenchymal transition of hepatocellular carcinoma by inhibiting YAP

**DOI:** 10.18632/oncotarget.20909

**Published:** 2017-09-15

**Authors:** Shuo Yu, Li Jing, Xiao-Ran Yin, Min-Cong Wang, Yi-Meng Chen, Ya Guo, Ke-Jun Nan, Li-Li Han

**Affiliations:** ^1^ Department of Oncology, The Second Affiliated Hospital of Xi’an Jiaotong University, Xi’an, Shaanxi Province 710004, China; ^2^ Department of Oncology, The First Affiliated Hospital of Xi’an Jiaotong University, Xi’an, Shaanxi Province 710061, China; ^3^ Department of General Surgery, The Second Affiliated Hospital of Xi’an Jiaotong University, Xi’an, Shaanxi Province 710004, China; ^4^ Department of Engineering Research Center of Bio-diagnosis and Biotherapy, The Second Affiliated Hospital of Xi’an Jiaotong University, Xi’an, Shaanxi Province 710004, China

**Keywords:** hepatocellular carcinoma, microRNA, YAP, EMT

## Abstract

MiR-195, a novel cancer-related microRNA, was previously reported to play an important role in many malignancies. This study aimed to investigate the role of miR-195 mediated epithelial–mesenchymal transition (EMT) and the progression of hepatocellular carcinoma (HCC) as well as the underlying mechanisms. Our result demonstrated that miR-195 were significantly down regulated in HCC and its decreased expression is associated with poor clinical features of HCC patients. Oppositely, expression level of YAP was significantly higher in HCC tissues, and the level of YAP in metastatic tissues was significantly higher. We also found that a strong inversely association between low level expression of miR-195 and high level of YAP in HCC tissues. Notably, this study confirmed that miR-195, YAP and their combination were valuable predictors for the prognosis of HCC patients. We also explored that miR-195 inhibits HCC growth and metastatic capacity. Mechanistically, we confirm that miR-195 inhibits the migration, invasion and EMT of HCC cells by suppressing YAP. Lastly, we revealed YAP was not only the downstream of miR-195 in HCC, but also mediated the promoting effects of miR-195 on the metastasis and EMT of HCC cells. Taken together, miR-195 inhibits the metastasis and EMT in HCC by targeting YAP. MiR-195/YAP pathway may potentially act as novel biomarker and attractive therapeutic target in HCC.

## INTRODUCTION

Hepatocellular carcinoma (HCC) is the sixth most common malignancy worldwide, and ranks as the second leading cause of cancer mortality [1, 2]. Although early detection, effective monitoring and more treatment options have improved the survival of patients with HCC, the long term survival remains poor. High probability of metastasis and recurrence after surgical resection are main responsibility for the HCC-related deaths [3]. In addition, the molecular mechanisms underlying HCC metastasis and recurrence have not been thoroughly understood. Consequently, identifying the mechanisms determining HCC metastasis is essential improving outcomes.

Accumulating evidence suggests that the epithelial-mesenchymal transition (EMT), characterized by the loss of epithelial polarity and acquisition of mesenchymal phenotype, play a vital role in the progression of metastasis in various carcinomas [4-6]. Epithelial cells that undergo EMT acquire the ability of migration and invasion: they lose original characteristics of adhesion, easily migrate from original sites, and then invade the lymphatic or vascular systems [7]. The EMT is regulated by a complex network in HCC cells. Recently, several studies have demonstrated that microRNAs could regulate EMT as critical modulators [8, 9].

MicroRNAs, a class of highly conserved non-coding RNAs, act as post-transcriptional regulators by regulating their target gene expression and a wide range of physiological and pathological processes including cell differentiation, proliferation, apoptosis, invasion and migration [10-12]. MiR-195 belongs to miR-15 family. In the past few years, there is a growing research focus on the function of miR-195 in tumorigenesis. MiR-195 has been known to act as a tumor suppressor in gastric cancer, breast cancer, thyroid cancer and some other types of cancers [13-15]. Further, some other studies suggested that miR-195 functioned as a metastatic inhibitor to suppress prostate cancer, osteosarcoma and NSCLC cells migration and invasion [16-18]. Although Wang et al. [19] reported that miR-195 was contributes to the lung metastasis of HCC. However, further investigations on the exact mechanisms of miR-195 function on HCC progression are still needed. Recently, Sun et al. [20] demonstrated that miR-195 was a potent suppressor of YAP1 which can be potential therapeutic targets as the chief downstream effectors of the Hippo pathway in a variety of cancers [21, 22]. Further, emerging reports demonstrated that YAP was involved in EMT in varies types of cancer, including hepatocellular carcinoma. YAP expression in human HCC cell lines is closely related with the characteristic markers of EMT, N-cadherin and E-cadherin expression [23]. Thus, we believe miR-195 may act an important function in HCC progression via inhibiting Hippo/YAP pathway.

In this study, we provide the first evidence that miR-195 can reverse EMT in HCC via inhibiting YAP. Our results demonstrated that miR-195 were significantly down regulated in HCC and its decreased expression is associated with poor clinical features of HCC patients. Oppositely, expression level of YAP was significantly higher in HCC tissues, and the level of YAP in metastatic tissues was significantly higher. We also found that a strong inversely association between low level expression of miR-195 and high level of YAP in HCC tissues. Notably, this study confirmed that miR-195, YAP and their combination were valuable predictors for the prognosis of HCC patients. We also explored that miR-195 inhibits HCC growth and metastatic capacity. Mechanistically, we confirm that miR-195 inhibits the migration, invasion and EMT of HCC cells by suppressing YAP. Lastly, we revealed YAP was not only the downstream of miR-195 in HCC, but also mediated the promoting effects of miR-195 on the metastasis and EMT of HCC cells. Taken together, this study demonstrates that miR-195/YAP pathway may potentially act as novel biomarker and attractive therapeutic target in HCC.

## RESULTS

### Down-regulation of miR-195 and up-regulation of YAP correlates with progression of human HCC

**Figure 1:**
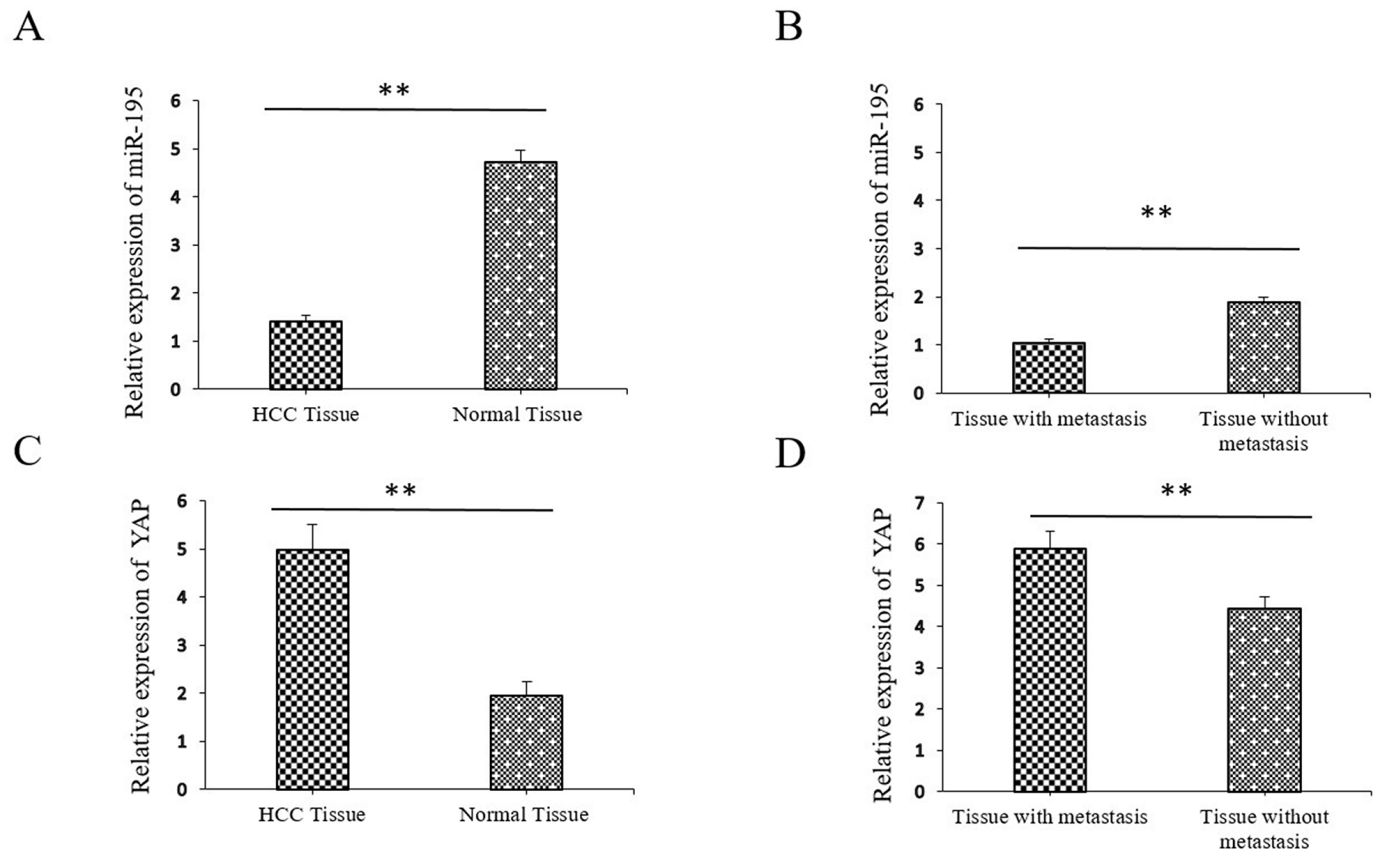
Expression of miR-195 and YAP in hepatocellular carcinoma (HCC) tissues compared to metastatic and paired distant non-cancerous tissues using qRT-PCR **(A)** The relative expression of miR-195 mRNA in HCC compared to distant non-cancerous HCC tissues (^**^P<0.01). **(B)** The relative expression of miR-195 mRNA in non-metastatic HCC tissues compared to metastatic HCC tissues (^**^P<0.01). **(C)** The relative expression of YAP mRNA in HCC compared to distant non-cancerous HCC tissues (^**^P<0.01). **(D)** The relative expression of YAP mRNA in non-metastatic HCC tissues compared to metastatic HCC tissues (^**^P<0.01).

We examined the expression of miR-195 in 130 randomly selected pairs of HCC tissues and adjacent non-tumor tissues. Specifically, qRT-PCR analysis showed that miR-195 were significantly lower in the HCC tissues than those in the distant non-cancerous tissues (1.41±0.14 vs. 4.72±0.28, p<0.01, Figure 1A). Moreover, we compared the expression level of miR-195 between metastatic and non-metastatic HCC tissues. The level of miR-195 in metastatic tissues was significantly lower than that in non-metastatic tissues. (1.03±0.09 vs. 4.89±0.11, p<0.01, Figure 1B). Oppositely, qRT-PCR analysis showed at the average expression level of YAP mRNA was significantly higher in HCC tissues than that in distant non-cancerous tissues (4.97±0.54 vs. 1.95±0.29, p<0.01, Figure 1C). Similarly, the level of YAP in metastatic tissues was significantly higher than that in non-metastatic tissues. (5.89±0.43 vs. 4.47±0.29, p<0.01, Figure 1D).

In addition, we further evaluated their clinical significance in HCC patients. As shown in Table 1, the lower miR-195 expression levels correlated with a larger tumor size (p=0.025), microscopic vascular invasion (p=0.027) and an advanced TNM stage HCC (p<0.001). Meanwhile, increased expression of YAP was correlated with larger tumor size (p=0.002), high level of AFP (p=0.024), microscopic vascular invasion (p<0.001) and advanced TNM stage (p<0.001) (Table 1). These results indicate that aberrant expression of miR-195 and YAP is correlated with the metastasis and progression of HCC.

**Table 1:** Association between miR-195 and YAP expressions and clinicopathological features in HCC

Variable	Total no. of patients n=130	miR-195		p	YAP		p
		Low expression	High expression		Low expression	High expression	
**Age (years)**				0.352			0.938
<50	54(41.5%)	39	15		16	38	
≥50	76(58.5%)	49	27		23	53	
**Gender**				0.778			0.924
Female	26(20.0%)	17	9		8	18	
Male	104(80.0%)	71	33		31	73	
**HBsAg**				0.406			0.955
Positive	113(86.9%)	75	38		34	79	
Negative	17(13.1%)	13	4		5	12	
**AFP (ng / mL)**				0.401			0.024^*^
<400	81(62.3%)	57	24		30	51	
≥400	49(37.7%)	31	18		9	40	
**Cirrhosis**				0.644			0.661
Yes	83(63.8%)	55	28		26	57	
No	47(36.2%)	33	14		13	34	
**Tumor size (cm)**				0.025^*^			0.002^**^
<5	53(40.8%)	30	23		24	29	
≥5	77(59.2%)	58	19		15	62	
**Tumor multiplicity**				0.477			0.431
Single	80(61.5%)	56	24		26	54	
Multiple	50(38.5%)	32	18		13	37	
**Differentiation**				0.394			0.538
Well–moderate	58(44.6%)	37	21		19	39	
Poor–undifferentiation	72(55.4%)	51	21		20	52	
**Microscopic vascular invasion**				0.027^*^			<0.001^**^
Yes	39(30.0%)	21	18		24	15	
No	91(70.0%)	67	24		15	77	
**Edmondson–Steiner grade**				0.819			0.072
I-II	42(32.5%)	29	13		17	25	
III-IV	88(67.5%)	59	29		22	66	
**Stage**				<0.001^**^			<0.001^**^
I-II	79(60.8%)	43	36		34	45	
III-IV	51(39.2%)	45 45	6		5	46 46	

^*^ p<0.05. ^**^ p<0.01.

### Association between expressions of miR-195 and YAP or EMT-related proteins in HCC tissue specimens

To determine the association between miR-195 with YAP expression in these 130 HCC specimens, expression levels were divided into four groups: miR-195(high)/ YAP (low), miR-195(high)/ YAP (high), miR-195(low)/ YAP(low) and miR-195(low)/ YAP (high). Notably, there are 68 tissues with miR-195(low)/ YAP (high) in the 130 HCC patients. We found a strong inversely association between low level expression of miR-195 and high level of YAP in HCC tissues (r=-0.230, p=0.009; Table 2). A statistically significant inversely correlation was not found between miR-195 and YAP expression in distant normal tissues (r=-0.070, p>0.05; Table 2).

**Table 2:** Association between miR-195 and YAP or between miR-195 and EMT-related protein expressions in HCC tissue specimens

199 Variable	miR-195		r	p
	High expression	Low expression		
**YAP**				
**Tumor**				
High expression	23	68	-0.230	0.009^**^
Low expression	19	20		
**Normal**			-0.070	0.427
High expression	27	21		
Low expression	53	29		
**Tumor**				
**E-cadherin**			0.532	p<0.001^**^
High expression	27	10		
Low expression	15	78		
**N-cadherin**			-0.388	p<0.001^**^
High expression	19	73		
Low expression	23	15		
**Vimentin**				
High expression	25	71	-0.211	0.016^*^
Low expression	17	17		

^*^ p<0.05. ^**^ p<0.01.

Next, we determined the association between the expression of miR-195 and EMT markers, such as E-cadherin, N-cadherin, and vimentin in HCC tissues. We found that low miR-195 expression was strongly associated with low E-cadherin expression (P<0.001, r=0.532), high N-cadherin expression (P<0.001, r=-0.388), and high vimentin expression (P=0.016, r=-0.211; Table 2). We also found that high YAP expression was strongly associated with low E-cadherin expression (P=0.006, r=-0.239), high N-cadherin expression (P<0.001, r=0.354), and high vimentin expression (P<0.001, r=0.304; Table 3).

**Table 3:** Association between YAP and EMT-related protein expressions in HCC tissue specimens

Variable	YAP		r	p
	High expression	Low expression		
**E-cadherin**			-0.239	P=0.006^**^
High expression	15	22		
Low expression	76	17		
**N-cadherin**			0.354	p<0.001^**^
High expression	74	18		
Low expression	17	21		
**Vimentin**				
High expression	72	24	0.304	p<0.001^**^
Low expression	19	15		

^*^ p<0.05. ^**^ p<0.01.

### Prognostic significance of differentially expressed miR-195 and YAP in HCC patients

**Figure 2:**
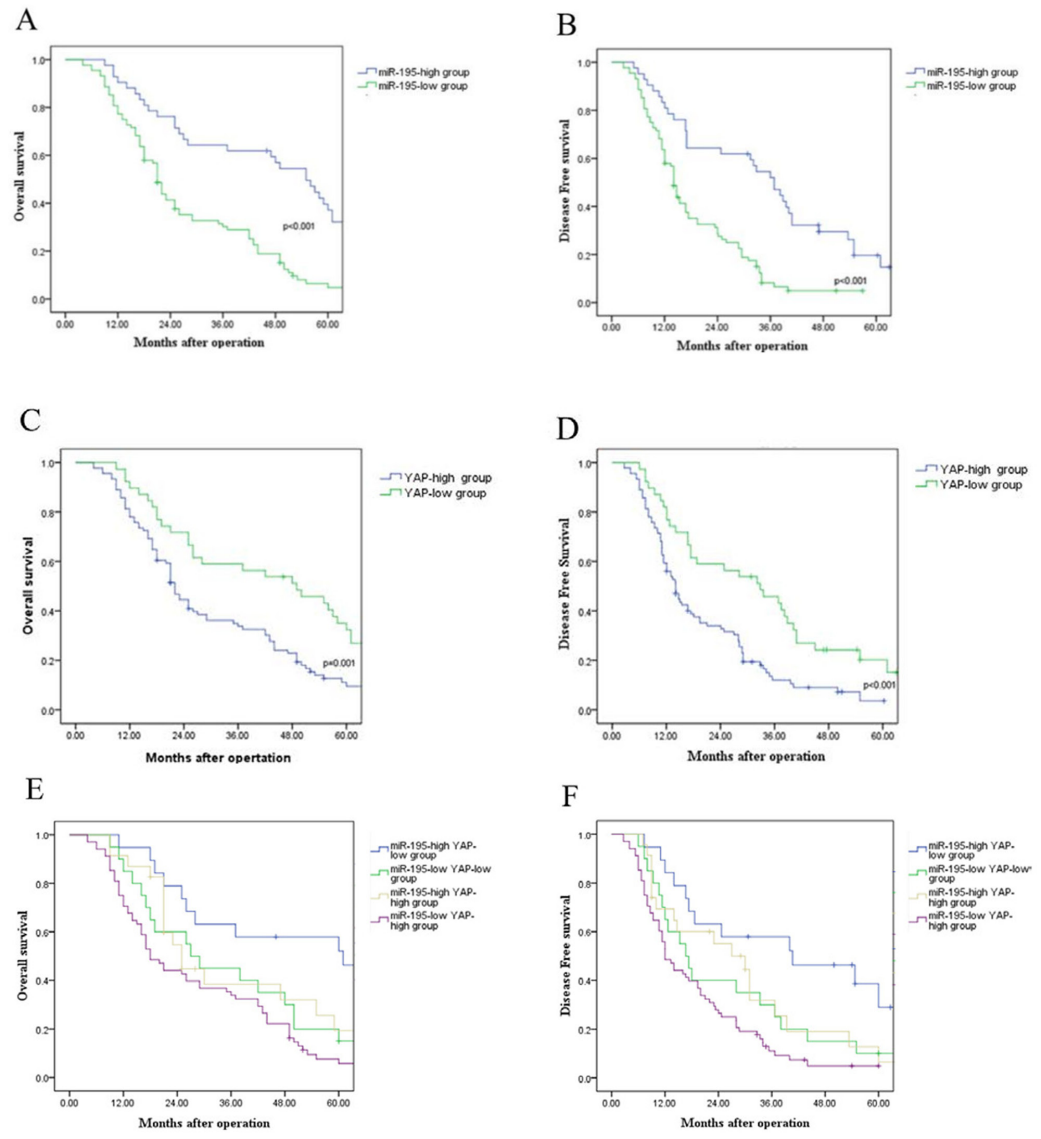
Prognostic value of miR-195 and YAP for HCC patients assessed by Kaplan–Meier analysis **(A)** Overall survival and **(B)** disease free survival were compared between HCC patients with high expression level of miR-195 and those with low level of miR-195. **(C)** Overall survival and **(D)** disease free survival were compared between HCC patients with high expression level of YAP and those with low level of YAP. **(E)** Overall survival and **(F)** disease free survival were compared between four subgroup of HCC patients (subgroup I: miR-195(high)/ YAP (low); subgroup II: miR-195(low)/ YAP(low); subgroup III: miR-195(high)/ YAP (high); subgroup IV: miR-195(low)/ YAP (high) (^**^P<0.01).

In this study, we investigated the prognostic value of miR-195 and YAP for HCC patients. Compared with those with high level of miR-195, patients with low expression of miR-195 had shorter overall survival (OS) (P<0.001, Figure 2A) and disease free survival (DFS) (P<0.001, Figure 2B). Otherwise, OS and DFS for patients with high YAP expression level were significantly worse than those for patients with low expression of YAP (P<0.001, respectively, Figure 2C and 2D). Furthermore, combination analysis showed that patients with miR-195(low)/ YAP (high) had the lowest OS and DFS (Figure 2E and 2F). In contrast, HCC patients with miR-195(high)/ YAP (low) had the most favorable OS and DFS. (Figure 2E and 2F). Consequently, our results indicate that combination of miR-195 and YAP can be an independent prognostic predictor for OS and DFS in HCC.

### MiR-195 expression affected the growth and invasion capacity of HCC cells

**Figure 3:**
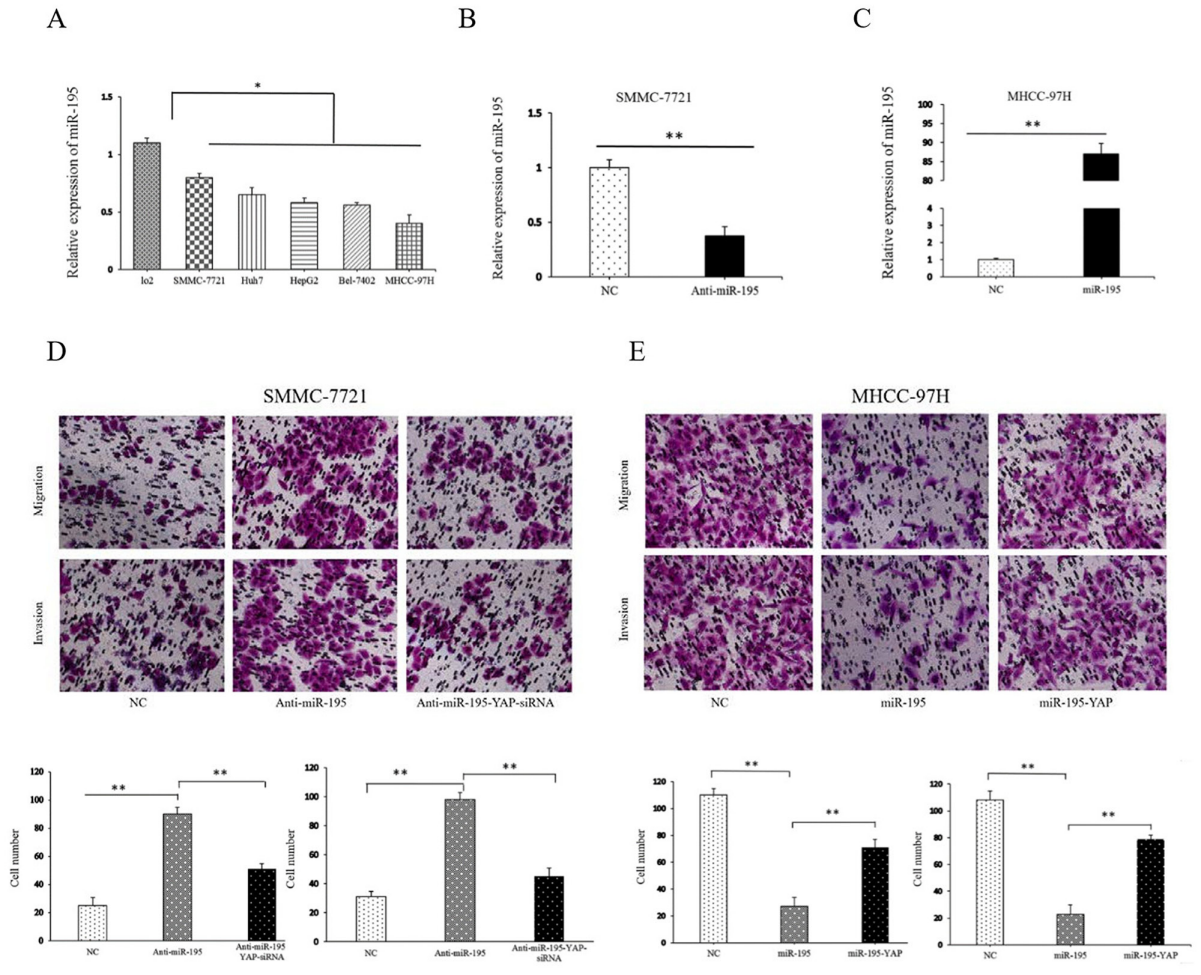
MiR-195 expression affected the invasion and migration capacity of HCC cells **(A)** The expression levels of miR-195 in various human HCC cell lines and human hepatocyte LO2 cells (^*^P<0.05). **(B)** The expression level of miR-195 was significantly decreased after transfection of miR-195 inhibitor into SMMC-7721 cells (^**^P<0.01). **(C)** The expression level of miR-195 was significantly increased after transfection of miR-195 mimics into MHCC-97H cells (^**^P<0.01). **(D, E)** Transwell tumor cell invasion assay. The number of invaded cells was quantified by counting the total numbers of cells from10 random fields (magnification, 200×). Data are presented as mean±SD of three in dependent experiments.

To assess the role of miR-195 in the development and progression of HCC, we determined the expression level of miR-195 in HCC cell lines and human liver cell line LO2. The results showed that miR-195 level was significantly decreased in HCC cell lines compared with LO2 cells (P<0.05, Figure 3A), which was consistent with the results of HCC tissues. Among HCC cell lines, the expression of miR-195 was highest in SMMC-7721 cells and weakest in MHCC-97H cells. Thus, we transfected SMMC-7721 cells with miR-195 inhibitor and MHCC-97H cells with miR-195 mimic. Compared with control cells, transfection of miR-195 inhibitor markedly reduced the expression level of miR-195 in SMMC-7721 cells (P<0.01, Figure 3B). Functionally, the transwell assay demonstrated that down-regulation of miR-195 resulted in significantly increased ability of the migration and invasion of SMMC-7721 cells (P<0.01, Figure 3D).

On the other hand, the expression level of miR-195 was significantly higher in MHCC-97H cells than that in control cells after transfection of miR-195 mimic (P<0.05, Figure 3C). Consequently, forced expression of miR-195 resulted in decreased migration (P<0.01, Figure 3E) and invasive ability of MHCC-97H cells significantly.

**Figure 4:**
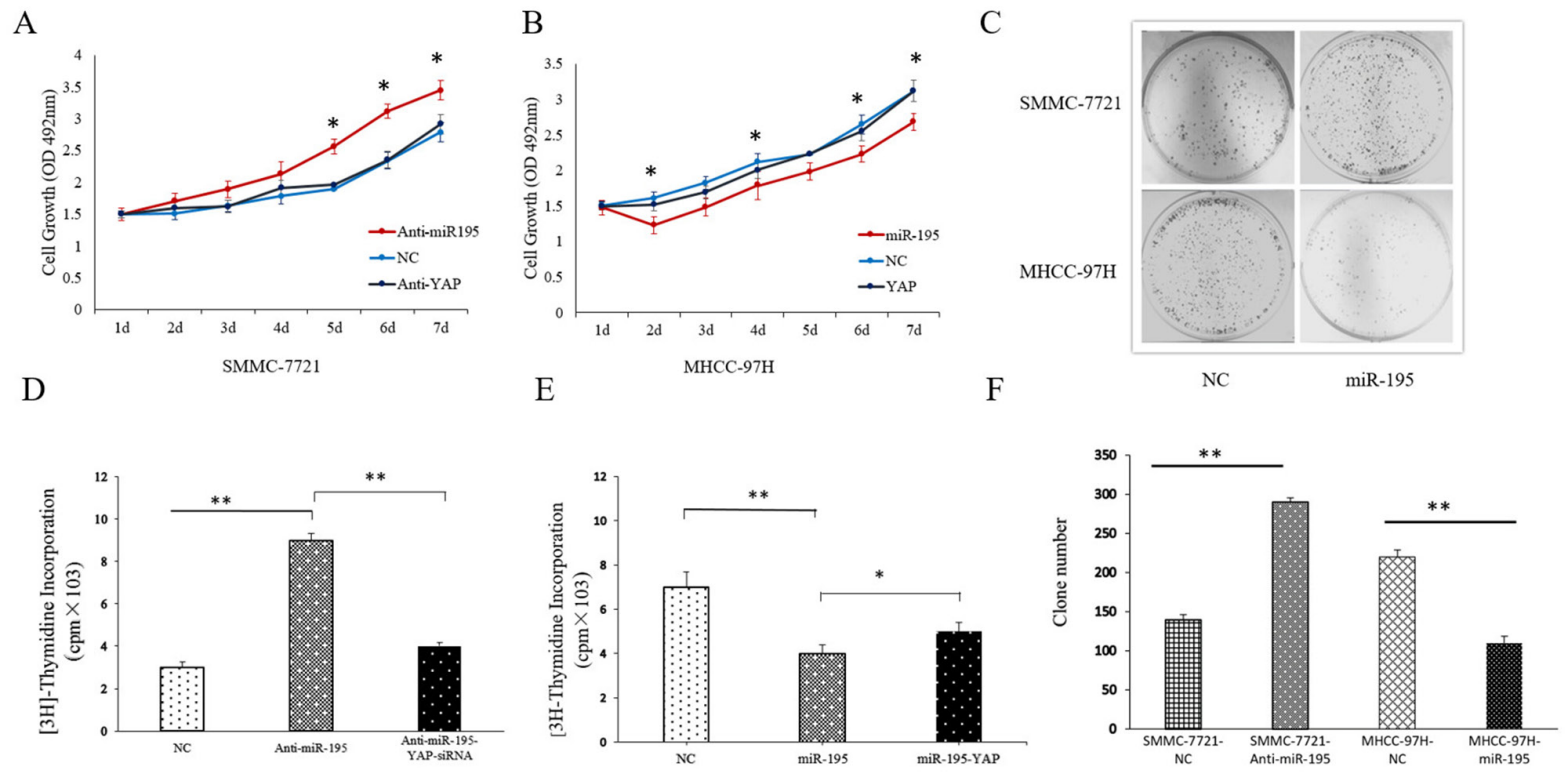
MiR-195 expression affected the proliferation and colony forming capacity of HCC cells **(A)** MTT assay showed cell proliferation ability of SMMC-7721 cells transduced with miR-195 inhibitor or transduced with miR-195 inhibitor and YAP siRNA (^*^P<0.05). **(B)** MTT assay showed cell proliferation ability of MHCC-97H cells after overexpressing miR-195 or transduced with miR-195 mimic and YAP vector (^*^P<0.05). **(C, F)** Colony formation assay showed inhibiting miR-195 significantly increased cell colony forming ability of SMMC-7721 cells while overexpression of miR-195 remarkably decreased cell colony forming ability of MHCC-97H cells (^**^P<0.01). **(D)** Proliferation effect of miRNA-195 and YAP on DNA synthesis in SMMC-7721. **(E)** Proliferation effect of miRNA-195 and YAP on DNA synthesis in MHCCC-97H.

Furthermore, we assessed cell proliferation and colony forming ability after altering the expression of miR-195 in HCC cells. The results of MTT and [^3^H]-thymidine incorporation into DNA assay showed that the proliferation ability of SMMC-7721 cells was stronger after inhibiting miR-195 (Figure 4A and 4D). Similarly, the colony forming assay revealed down-regulation miR-195 also resulted in the increased colony forming ability of SMMC-7721cells. (Figure 4C). In contrast, overexpressing miR-195 resulted in decreased proliferation and colony forming ability of MHCC-97H cells significantly (Figure 4B and 4C).

### MiR-195 inhibits EMT of HCC cells

**Figure 5:**
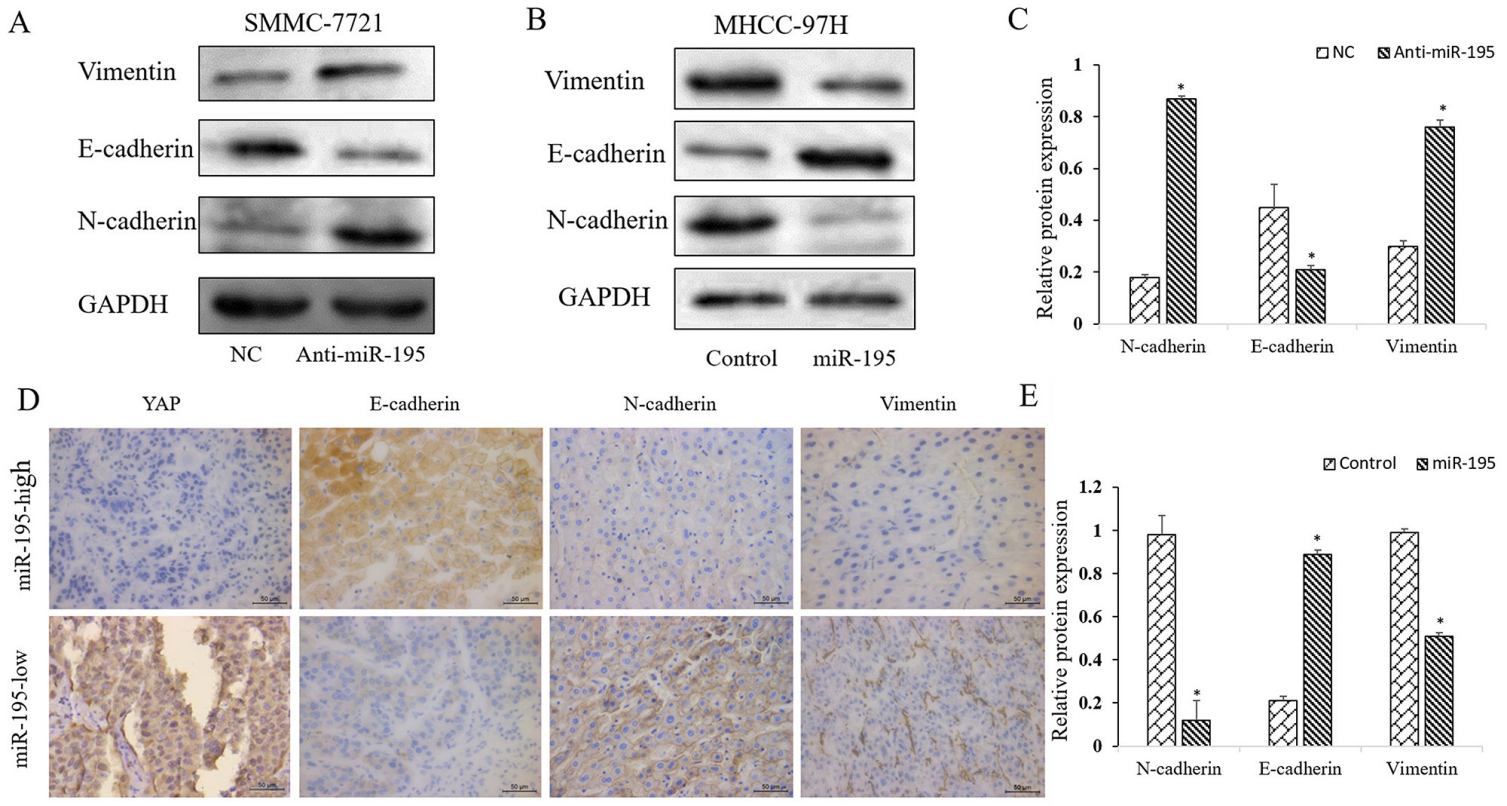
MiR-195 inhibits EMT of HCC cells **(A-C)** Western blot analysis of E-cadherin and vimentin after down-regulating miR-195 in SMMC-7721 cells and up-regulating miR-195 in MHCC-97H cells. GAPDH was used as an internal control (^*^P<0.05). **(D, E)** Immunohistochemistry of E-cadherin, N-cadherin and vimentin were showed and compared between tissues of high miR-195 level and those of low miR-195 level (^*^P<0.05). (400×).

Since EMT is a well-recognized process underlying the metastasis of HCC cells and micro-RNAs have been found to important regulator of EMT, we assessed whether miR-195 could modulate the EMT phenotype of HCC cells. WB results showed that the expression of E-cadherin was decreased while vimentin expression was increased after inhibiting miR-195 in SMMC-7721 cells (P<0.01, Figure 5A). In MHCC-97H cells, miR-195 mimics resulted in significantly increased expression of E-cadherin and decreased expression of vimentin (P<0.01, Figure 5B). To further confirm that miR-195 could inhibit the EMT of HCC, we examined the expression level of E-cadherin and vimentin in clinical HCC tissues using IHC. As shown in Figure 5D, the expression of E-cadherin was significantly lower (P<0.01) and the expression of vimentin was significantly higher (P<0.01) in tissues with lower expression level of miR-195. These results indicate that miR-195 regulates the invasion and metastasis of HCC cells by inhibiting EMT.

### Modulation of miR-195 expression regulated YAP signaling pathway

**Figure 6:**
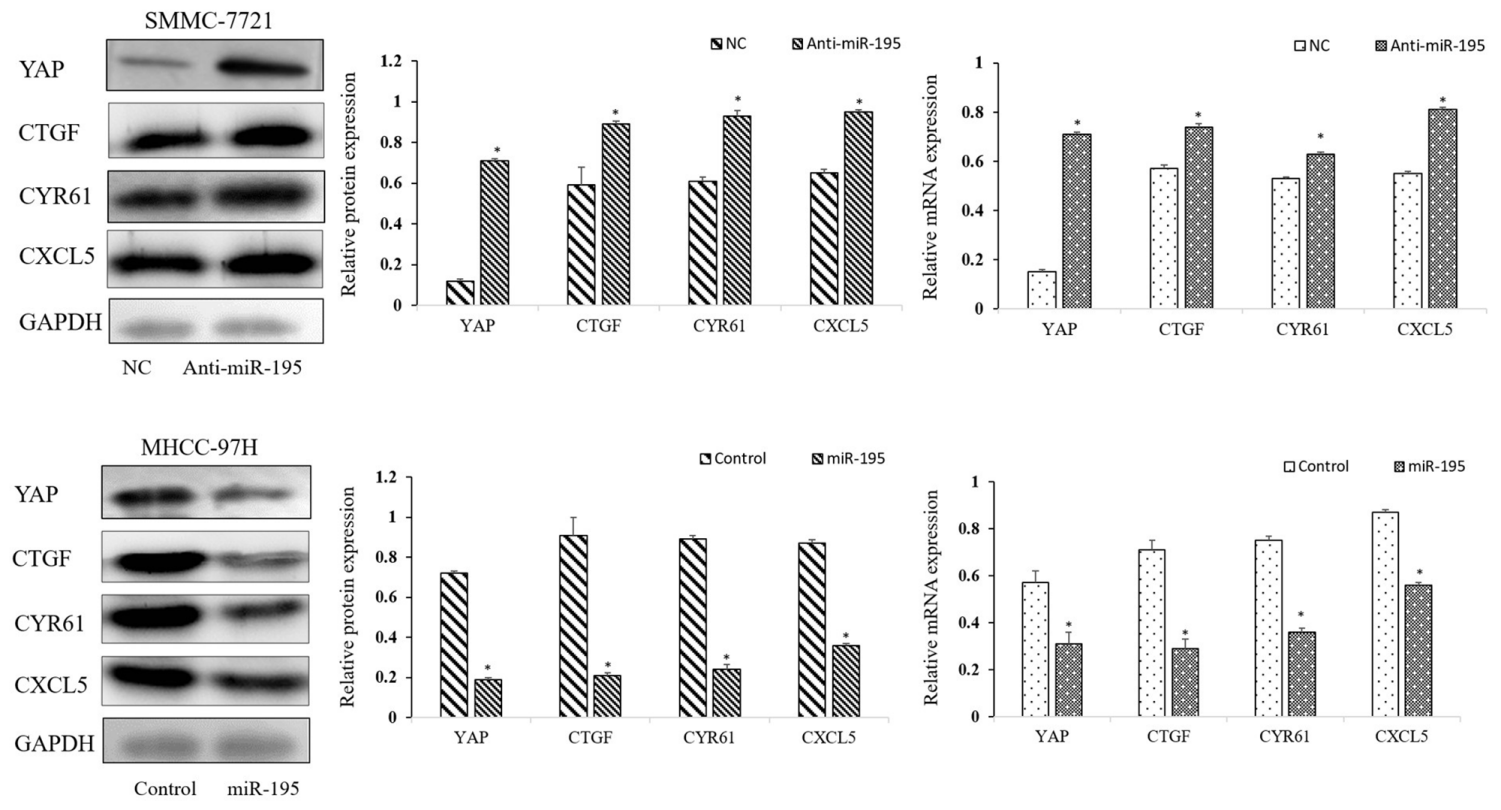
Modulation of miR-195 expression regulated YAP signaling pathway Down-regulation or overexpression of miR-195 regulated the mRNA and protein level of YAP and YAP pathway-related gene (^*^P<0.05).

Recently, miR-195 was demonstrated as a suppressor of Hippo-YAP pathway in colorectal cancer [20]. Our previous study also showed that YAP expression was negatively correlated with the expression of miR-195 in HCC tissues. Furthermore, Western-blot and qRT-PCR assay showed that knockdown of miR-195 expression significantly up-regulated the mRNA and protein level of YAP, compared to control cells. In contrast, up-regulation of miR-195 expression significantly decreased YAP and YAP/TEAD-regulated gene, including Connective Tissue Growth Factor (CTGF), Cysteine-rich angiogenic inducer 61 (CYR61) and CXCL5 (Figure 6).

To reveal whether miR-195 binds directly to the binding site in the YAP 3`-UTR, we searched public databases for the candidate targets of miR-195. We found that the 3`-UTR of YAP mRNA contained the complementary sequence of miR-195 (Figure 8A). We also performed dual-luciferase reporter gene assays to determine whether miR-195 could directly target 3`-UTR of YAP mRNA. As shown in Figure 8B, miR-195 inhibition significantly increased the luciferase activity of YAP containing a wild-type (wt) 3`-UTR in SMMC-7721 cells. And overexpression of miR-195 in MHCC-97H cells significantly suppressed the luciferase activity of wt 3`-UTR of YAP. However, altering miR-195 expression did not significantly influence the luciferase activity of YAP with a mutant (mt) 3`-UTR. Taken together, the data from both HCC cells and clinical samples revealed that YAP is a direct downstream target of miR-195 in HCC.

**Figure 7:**
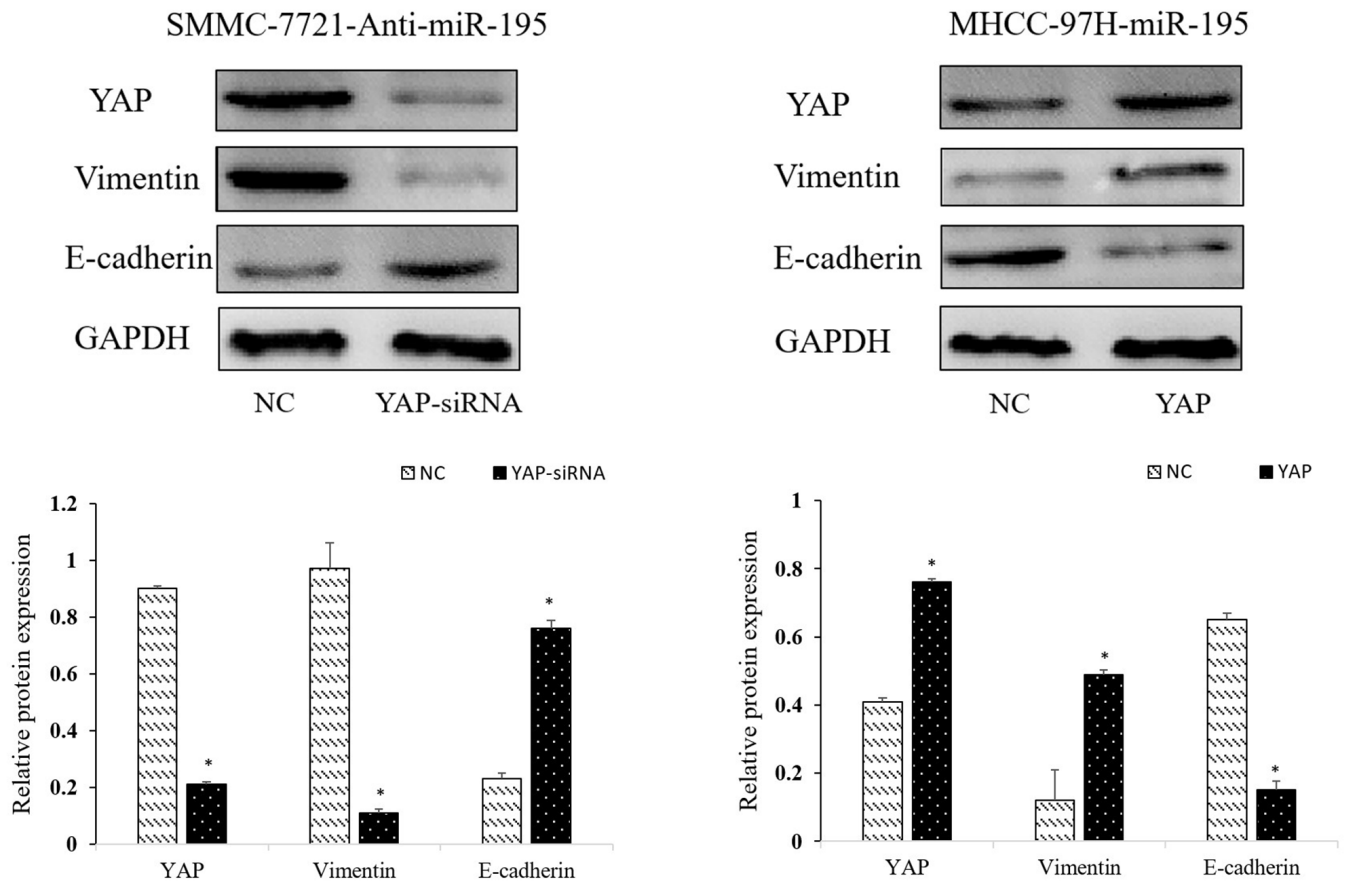
Repression of YAP is required for the inhibitory effects of miR-195 on metastasis and EMT of HCC cells Western-blot analysis of the YAP influence the expression of EMT-related proteins (E-cadherin, N-cadherin and Vimentin) (^*^P<0.05).

**Figure 8:**
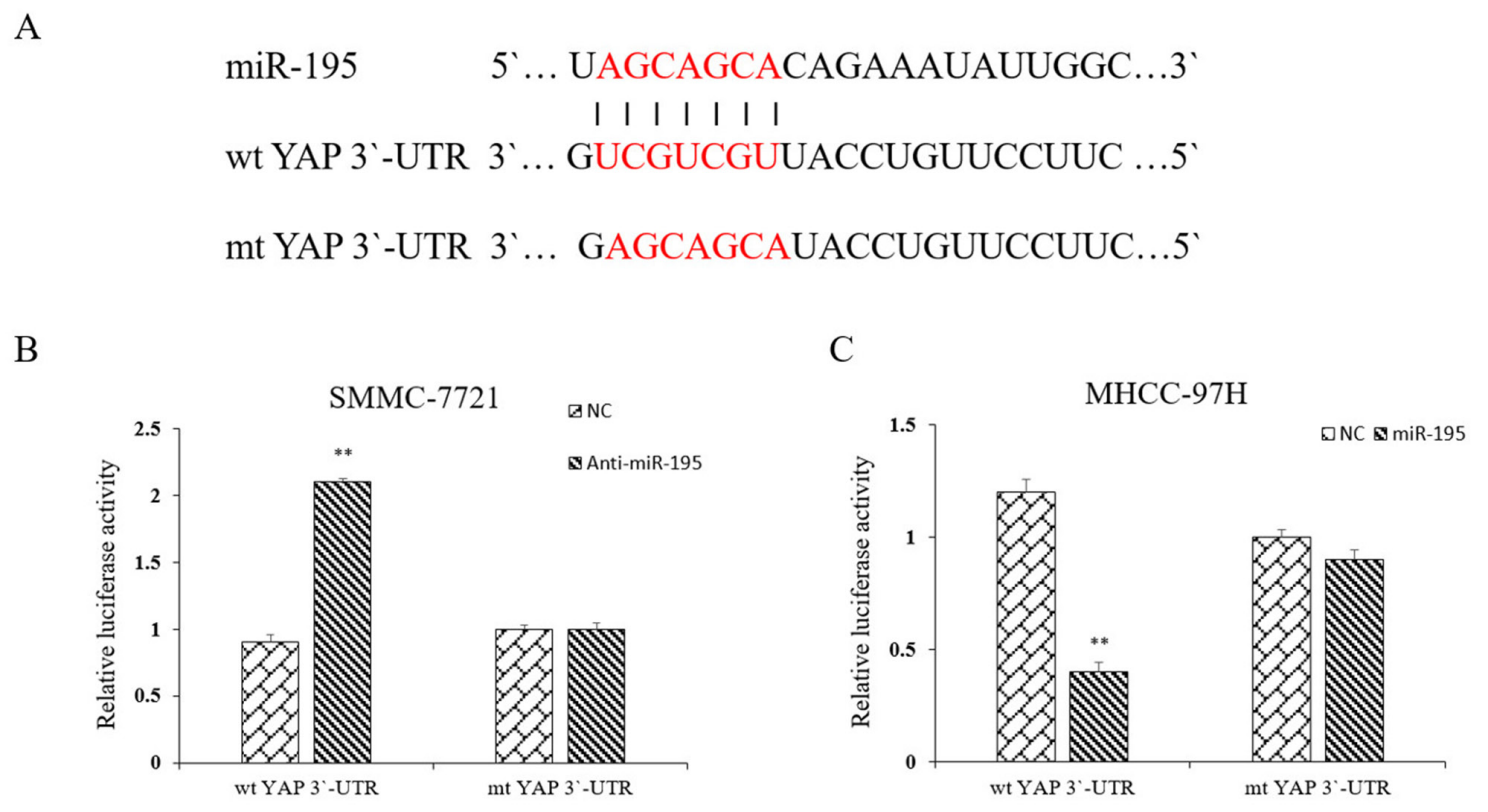
YAP is a direct downstream target of miR-195 in HCC **(A)** miR-195 and its putative binding sequence in the 3′-UTR of YAP. **(B)** Inhibition of miR-195 significantly increased the luciferase activity that carried wild type but not mutant type3′-UTR of YAP in MHCC-7721 cells (^**^P<0.01). **(C)** Overexpression of miR-195 significantly decreased the luciferase activity that carried wild type but not mutant type 3′-UTR of YAP in MHCC-97H cells (^**^P<0.01).

### The YAP signaling pathway modulated the effects of miR-195 on HCC proliferation, invasion and EMT

After confirming miR-195 could regulate YAP expression in HCC, we further explored the role of YAP in the biological function of miR-195 in HCC. YAP siRNA significantly reduced YAP expression level in SMMC-7721 transfected with miR-195 inhibitors; while YAP vector increased YAP expression level in MHCC-97H overexpressing miR-195 (P<0.05, Figure 7). Consequently, YAP knockdown reversed the epithelial transition induced by miR-195 inhibition in SMMC-7721 cells as suggested by the increased E-cadherin expression and decreased vimentin expression (P<0.05, Figure 7). Functionally, decreased YAP abrogated the promoting effects of miR-195 inhibition on the migration and invasion of SMMC-7721 cells (P<0.01, Figure 3D). On the other hand, increased YAP expression in MHCC-97H cells could promoted the EMT of MHCC-97H cells overexpressing miR-195, as suggested by decreased E-cadher and increased vimentin (P<0.05, Figure 7). And YAP overexpression abrogated the inhibitory effects of miR-195 overexpression on the migration and invasion of MHCC-97H cells (P<0.01, Figure 3E). MTT assay revealed altering YAP expression also reversed the proliferation effect induced by miR-195 inhibition or overexpressing miR-195((P<0.05, Figure 4A and 4B).

These data suggest that YAP is not only a downstream target of miR-195, but also a functional mediator of miR-195 in HCC.

## DISCUSSION

Emerging evidence has revealed that microRNAs play a crucial role in the development and progression of HCC [24-27]. MiR-195 have been proposed as novel tumor suppressor, effective metastatic inhibitor and attractive therapeutic targets in some types of cancers [16-18]. However, further investigations on the clinical significance functional effects and the molecular mechanisms of specific miR-195 in HCC are still needed. In our current study, the results in HCC tissues were consistent with data *in vitro*, adding novel information regarding miR-195 tumorigenesis.

Recent study showed that miR-195 significantly down-regulated in HCC tissues contributed to the lung metastasis of HCC [19]. Moreover, miR-195 was demonstrated as a suppressor of Hippo-YAP pathway in colorectal cancer [20]. In this study, we confirmed that expression of miR-195 was significantly down-regulated in HCC tissues, and especially in the tissues of patients who have metastasis. Oppositely, expression level of YAP was significantly higher in HCC tissues, and the level of YAP in metastatic tissues was significantly higher. Moreover, we demonstrated for the first time the strong inversely association between low level expression of miR-195 and high level of YAP in HCC tissues. These indicate that miR-195 maybe involved in the regulation of YAP/TEAD signal way. A group of independent studies confirmed that microRNAs could act as valuable biomarkers for the early diagnosis and prognostic prediction in HCC [28-30]. In this study, we also revealed that the lower miR-195 expression levels significantly correlated with unfavorable clinical features of HCC patients. Meanwhile, increased expression of YAP was associated with adverse clinical features of HCC patients. These strongly suggest that aberrant expression of miR-195 and YAP is correlated with the metastasis and progression of HCC. Furthermore, survival analysis confirmed miR-195, YAP and their combination significantly associated with the prognosis of HCC patients. Interestingly, HCC patients with low expression of miR-195 and high expression of YAP had the lowest OS and DFS. These results strongly indicate that miR-195, YAP and their combination were valuable predictors for the prognosis of HCC patients.

Recurrence or metastasis of HCC is mainly culprit of the poor prognosis of HCC patients [3]. MicroRNAs have been found to serve as an important role in modulating the invasion and metastasis of human cancer [31, 32]. Therefore, we explored whether miR-195 could participate in the progression of HCC by regulating the metastatic ability of HCC cells. Our results showed that overexpression of miR-195 decreased the metastatic ability of HCC cells while down-regulation of miR-195 increased the metastatic ability of HCC cells. EMT has been demonstrated as the critical mechanism for the invasion and metastasis of cancer cells, which was characterized as decreased expression of epithelial marker and increased level of mesenchymal markers [6]. Accumulating evidences have demonstrated that microRNAs play a critical role in the progression of EMT in HCC [33-36]. It has been reported previously that miR-195 has an influence on EMT in prostate cancer cells [16]. In the current study, we found that down-regulation of miR-195 decreased E-cadherin expression and increased N-cadherin and vimentin expression, whereas increased expression of miR-195 showed opposite effects. Furthermore, IHC results of HCC tissues further confirmed that HCC tissues with low level of miR-195 had significantly lower level of E-cadherin and higher level of N-cadherin and vimentin. These data suggest that miR-195 could inhibit the metastasis of HCC by suppressing EMT of HCC, and could act as a potentially therapeutic target of HCC metastasis.

YAP has been found to be overexpressed in human cancers and promotes the growth, proliferation and invasion of cancer cells [37-41]. Furthermore, other studies suggested YAP also had an important influence on EMT in breast cancer [42], pancreatic cancer [43], colorectal cancer [44] and hepatocellular carcinoma [23]. Here, the expression of YAP was inversely correlated with the expression level of miR-195 in HCC tissues. And inhibition of miR-195 significantly increased YAP, while overexpression of miR-195 remarkably decreased the expression of YAP in HCC cells, indicating YAP was under the regulation of miR-195. In addition, our study also have shown that the expression of YAP was inversely correlated with the expression of E-cadherin in HCC tissues, supporting the finding that YAP could promote the metastasis and EMT of HCC. In this study, we further demonstrated YAP was not only the downstream of miR-195 in HCC, but also mediated the promoting effects of miR-195 on the metastasis and EMT of HCC cells. Altering YAP expression could reverse the influence of miR-195 on the migration, invasion and EMT of HCC cells. In all, these data suggest that miR-195/YAP pathway exerts important role inhibiting the metastasis and EMT of HCC.

However, it is necessary to mention here that the molecular mechanisms by which YAP contributes to the EMT of HCC is still unclear. Interestingly, YAP was found to modulate TGF-β1-induced simultaneous apoptosis and EMT through up-regulation of the EGF receptor in mouse mammary epithelial (NMuMG) cells [45]. And previous studies showed that TGF-β1could promote the metastasis and EMT of HCC. Therefore, YAP could probably influence the EMT of HCC by modulating TGF-β1. The detailed mechanisms underlying the promoting effects of YAP on EMT of HCC are worth to be investigated in detail in the future.

In conclusion, the current study confirmed that miR-195 is down-regulated in HCC and its decreased expression is associated with poor clinical features of HCC patients. Notably, miR-195, YAP and their combination are promising prognostic predictors for the survival of HCC patients. We also explored that miR-195 inhibits HCC growth and metastatic capability. Mechanistically, we confirm that miR-195 inhibits the migration, invasion and EMT of HCC cells by suppressing YAP. Furthermore, we revealed YAP was not only the downstream of miR-195 in HCC, but also mediated the promoting effects of miR-195 on the metastasis and EMT of HCC cells. Taken together, this study demonstrates that miR-195/YAP pathway may potentially act as novel biomarker and attractive therapeutic target in HCC.

## MATERIALS AND METHODS

### Patients and tissue samples

This study was approved by the Ethics Committee of Clinical Research of The First Affiliated Hospital, Medical College, Xi’an Jiaotong University (Shannxi, China). All patients provided written informed consent. Tissue specimens were obtained from 130 patients who underwent surgical resection of HCC lesions at The First Affiliated Hospital, College of Medicine of Xi’an Jiaotong University between January 2010 and January 2011. None of the patients had received prior radiotherapy or chemotherapy. All patients were diagnosed histologically (Table 1). Paraffin tissue blocks were retrospectively retrieved, which contained both cancerous and distant non-cancerous tissues. Fresh tissues were obtained during surgery, immediately snap-frozen in liquid nitrogen, and stored at −80°C until use.

### Immunohistochemistry

Antibodies included a rabbit polyclonal anti-YAP anti body at a dilution of 1:50, anti-E-cadherin antibody at a dilution of 1:100, anti-N-cadherin antibody at a dilution of 1:100, and anti-vimentin antibody at a dilution of 1:100 (Beijing Biosynthesis Biotechnology, China). These antibodies were specific for immunohistochemistry. The negative control sections were incubated with phosphate buffered saline (PBS) to replace the primary antibody. Three pathologists reviewed the immunostained sections under a light microscope and scored them in 10 randomly selected ×20 power fields. The staining intensity was graded as: 0, no staining; 1, weak; 2, moderate; and 3, strong. The percentage of positive cells was scored as: 1, <25%; 2, 26-50%; 3, 51-75%; and 4,>76%. These two scores were added together, and each tissue sample was categorized into four groups: 0,≤5% cells were stained; 1-3, weak expression; 4-5, moderate expression; and 6-7, strong expression. Finally, the number of cells with low-to weak expression and the number of cells with moderate-to-strong expression were compared statistically.

### Quantitative reverse transcriptase polymerase chain reaction (qRT-PCR)

The primers were designed and synthesized by Takara (Dalian, China) and GAPDH was used as the internal control. Each measurement was performed in triplicate and repeated twice. The level of U6 RNA was measured and used to normalize the relative abundance of miR-195. Expression levels of YAP and GAPDH mRNA were evaluated using a relative quantification approach (2–ΔΔCt method) against GAPDH levels.

### Cell lines, culture and transfection

HCC cell lines (LO2, SMMC-7721, Huh7, HepG2, Bel-7402 and MHCC-97H) were obtained from American Type Culture Collection (ATCC; Manassas, VA, USA) and cultured in specific culture medium according to ATCC. All cells were cultured in complete Dulbecco’s modified Eagle medium (DMEM, Gibco, Grand Island, NY, USA) containing 10% fetalbovine serum (FBS, Gibco) with100 units/mL penicillin and 100μg/mL streptomycin (Sigma, St-Louis, MO, USA) in a humidified air containing 5% CO2 at 37°C.

The miR-195 mimic, a control vector were all purchased from Genecopoeia (Guangzhou, China). These miRNA vectors were transfected into HCC cells using Lippofectamine 2000 based on manufacture’s protocol. The YAP plasmid DNA used to over-express the YAP gene in the cells was purchased from Addgene (Cambridge, MA). YAP specific siRNA was bought from Origene (Beijing, China). YAP expressing vector or YAP siRNA was transfected into HCC cells with Lipofectamine 200 following manufacturer’s protocol to overexpress or downregulate YAP in corresponding HCC cells.

### Western blotting

For protein extraction, the cell lines were lysed by using M-PER Mammalian Protein Extraction Reagent (Thermo) supplied with Complete Protease Inhibitor Cocktails (Roche, Lewes, UK). The total amount of protein for each sample was 20μg, and the samples were run on 4∼20% gradient SDS–polyacrylamide gels (Bio-Rad Laboratories, Inc., Hercules, CA) and then were transferred to Immobilon-Pnitrocellulose membranes (Millipore, Bellerica, MA). The membranes were probed with primary antibodies YAP, N-cadherin, E-cadherin, Vimentin, CTGF, CYR61, CXCL5(Cell Signaling Technology, Inc) and GAPDH (Sigma-Aldrich) in 4°C overnight after being blocked with 5% non-fat milk. The membranes were then incubated with appropriate second antibodies, as well as anti-rabbit bodies for YAP, N-cadherin, E-cadherin, Vimentin, CTGF, CYR61, CXCL5 and anti-mouse antibody for GAPDH at room temperature for 1 hour, and finally were detected by using an ECL blotting analysis system (Amersham Pharmacia Biotech, Piscataway, NJ) Colony formation assay.

### Matrigel invasion assay

We used 24-well plates inserted with 8.0μm pore Transwells (Millipore), and the membranes of the inserts were pre-coated with 15μg of Matrigel (Becton Dickinson Bioscience, Bedford, MA, USA). Cells (2×10^4^) in 200μl growth media without FBS were placed onto the top chamber. Normal culture media was added to the bottom chamber. After 36 h of incubation, cells on the top surface of the membrane were removed with acottonswab, while invading cells adhered to the underside of the membrane were fixed with methanol, stained with crystal violet solution, and counted in 10 random fields under a microscope.

### Statistical analysis

Data were analyzed using one-way ANOVA or two-tailed Student’s t-tests for comparison between groups. Chi-square tests and Fisher’s exact tests were performed to assess association of clinicopathological parameters with protein expression. A Spearman test was used to analyze the correlation between gene expressions. Survival curves were plotted using the Kaplan–Meier method. Statistical Package for SocialScience (SPSS) version17.0 (SPSSInc., Chicago, IL) was used, and P<0.05with a two-sided test was considered statistically significant.

## Abbreviations

EMT: epithelial–mesenchymal transition
HCC: hepatocellular carcinoma
YAP: Yes-associated protein
CTGF: Growth Factor
CYR61: Cysteine-rich angiogenic inducer 61
OS: verall survival
DFS: disease free survival
